# Family protection motivation and economic vulnerability: a network analysis of public influenza risk perception, education and mitigation strategies in China

**DOI:** 10.3389/fpubh.2025.1633541

**Published:** 2025-08-08

**Authors:** Cheng Yang, Jiawei Li, Xianqiong Feng, Shaoyu Su, Qin Zeng

**Affiliations:** ^1^Department of Pediatric Intensive Care Unit Nursing, West China Second University Hospital, Sichuan University, Chengdu, China; ^2^Key Laboratory of Birth Defects and Related Diseases of Women and Children (Sichuan University), Ministry of Education, Chengdu, China; ^3^Department of Pediatric Outpatient Nursing, West China Second University Hospital, Sichuan University, Chengdu, China; ^4^Department of Nursing, West China Hospital, Sichuan University, Chengdu, China; ^5^West China School of Nursing, Sichuan University, Chengdu, China; ^6^Department of Pediatrics Nursing, West China Second University Hospital, Sichuan University, Chengdu, China

**Keywords:** influenza risk perception, network analysis, family protection motivation, socioeconomic vulnerability, science literacy, public health strategy, cross-sectional network analysis study using convenience sampling

## Abstract

**Background:**

In the post-pandemic era, influenza and COVID-19 jointly exacerbate global public health burdens, yet persistent biases in risk perception drive declining vaccination rates and health disparities. Conventional linear models fail to capture the complex interactions between risk cognition, family protection motivation, and socioeconomic vulnerability—particularly within collectivist contexts like China. This gap impedes effective interventions targeting critical behavioral nodes in influenza mitigation.

**Objective:**

This study employs network analysis to uncover the core structural features of influenza risk perception among the Chinese public, examining the association between science literacy and risk perception to inform targeted mitigation and intervention strategies.

**Design:**

A multicenter, cross-sectional network analysis study using convenience sampling.

**Setting:**

Fifteen provinces across mainland China, covering eastern, western, southern, northern, and central regions.

**Participants:**

1,416 individuals aged 18–70, representing diverse occupations, education levels, and income groups.

**Results:**

The public’s influenza risk perception network exhibited a “family-knowledge-economy” triadic structure. “Risk of family infection” (M_2) emerged as the central node (strength = 2.165), while “transmissibility knowledge” (F_3) and “socioeconomic loss” (S_2) served as the key knowledge nodes (strength = 1.520) and bridge node (bridge strength = 2.037). Additionally, science literacy moderated risk perception by enhancing perceived control, with the strongest association observed between knowledge level and “temporal controllability” (C_3, edge weight = 0.25). Family-based knowledge-sharing effects were significant (K_1-K_2 edge weight = 0.42). Network stability tests confirmed robustness (centrality stability coefficient CS > 0.5, core node differences *p* < 0.01).

**Conclusion:**

Network analysis reveals a “family-knowledge-economy” triad governing influenza risk perception, with family infection risk (M_2) as the central driver (strength = 2.165) and socioeconomic loss (S_2) as the pivotal bridge node (bridge strength = 2.037). Science literacy amplifies perceived controllability (C_3–K_1 edge = 0.25) but fails to alleviate economic anxiety, underscoring the need for integrated structural policies. Family-centered interventions—leveraging tiered communication, economic security narratives, and real-time surveillance of network dynamics—are essential to optimize public health strategies in collectivist societies.

## Introduction

Influenza continues to pose a formidable global public health challenge in the post-pandemic era, exhibiting pronounced seasonal epidemic patterns and persistent pandemic potential. According to WHO estimates, the disease causes approximately 1 billion annual cases worldwide, resulting in 3–5 million severe illnesses and 290,000–650,000 deaths (1, 2), with associated economic losses exceeding tens of billions of US dollars. Notably, socioeconomic determinants significantly influence disease burden, as demonstrated by global analyses revealing how financial development and emediates. The post-COVID-19 era has introduced new complexities, including increased risks of influenza virus recombination and subtype co-circulation (creating potential “twindemic” scenarios) (3, 4), further exacerbated by environmental co-factors such as seasonal air pollution that amplify respiratory mortality.

Despite extensive research, critical gaps remain in understanding the intricate mechanisms governing influenza risk perception. Current literature fails to adequately address this issue due to three interrelated methodological and theoretical limitations:

1. Overreliance on Linear Modeling Approaches: Conventional methods, particularly linear regression, inadequately capture the dynamic, non-linear interactions between cognitive dimensions (e.g., perceived severity and susceptibility) and contextual factors such as urbanization-driven health disparities or evolving socioeconomic stressors, ignores the systemic complexity (5–7). This static perspective ignores systemic complexity.
2. Inadequate Consideration of Post-Pandemic Contexts: Many studies neglect the profound psychosocial shifts following COVID-19, including widespread vaccine fatigue and heightened socioeconomic pressures that disproportionately affect vulnerable populations (8–10). These factors are essential for understanding contemporary risk assessment.
3. Theoretical Disintegration: Prevailing frameworks often dissociate individual risk perception from macro-level public health determinants, including energy transitions, environmental policies (e.g., air quality regulations), and systemic economic vulnerabilities (11–14). This disconnection impedes comprehensive understanding.

These limitations directly impact on health behaviors (e.g., vaccination hesitancy and suboptimal antiviral use (15, 16)), leading to systematic underestimation of disease severity and substantial miscalibration of hospitalization risks–problems further compounded by limited health literacy impairing risk assessment capabilities. The inability to model the interconnected system of cognitive, socioeconomic, and environmental factors dynamically shaping risk perception in the current era highlights the urgent need for novel approaches.

This study conceptualizes family protection motivation as an individual’s intrinsic drive to protect family members from health threats, particularly influenza. This motivation stems from concerns about intra-family disease transmission, where individuals assume responsibility for preventing infection spread within their household unit. It encompasses emotional concerns, protective behavioral intentions, and multidimensional risk perception (17). This operationalization is pivotal for understanding the psychosocial mechanisms underlying preventive health behaviors like vaccination adoption and hygiene compliance in familial contexts (18).

The theoretical framework builds upon the Extended Protection Motivation Theory (PMT), which postulates that protective behaviors emerge from dual appraisal processes: threat appraisal (perceived severity and vulnerability) and coping appraisal (response efficacy and self-efficacy) (19). Our extension incorporates two novel dimensions: (1) family-oriented protection motives that amplify threat appraisal in collectivist cultures (20), and (2) socio-economic vulnerabilities that modulate coping appraisal capacities (21). This adaptation addresses PMT’s Western-centric limitation by contextualizing protection motivation within China’s familial-social ecosystem (22).

While traditional models like the Health Belief Model (HBM) employ linear pathways between perceived susceptibility, severity, and behavioral outcomes, they fail to capture the complex, recursive nature of risk perception systems. Network analysis overcomes these limitations through three key advantages: (1) mapping bidirectional relationships between cognitive, behavioral, and contextual factors (23); (2) identifying system-critical nodes (e.g., family concerns as bridge elements between threat appraisal and economic constraints) (17); and (3) revealing non-linear dynamics where socio-economic vulnerabilities may paradoxically inhibit protective behaviors despite high threat perception (24). This systems-level approach provides unprecedented insights into how family protection motivations interact with structural constraints to shape pandemic preparedness behaviors.

This study addresses these limitations through three key innovations:

1. Network Psychometrics Framework: We pioneer the application of network analysis to conceptualize influenza risk perception as a dynamic, interconnected system. This approach surpasses traditional linear regression by elucidating complex interactions among cognitive, affective, and behavioral components, offering unprecedented insights into system structure and dynamics.
2. Integrated Multi-Scale Determinants: We innovatively incorporate family-level protection motivations, socioeconomic stress (economic vulnerability), household structure, and environmental co-exposures into a unified analytical framework, bridging the critical divide between micro-level cognition and macro-level structural determinants.
3. Identification of Critical Network Features: Our methodology uniquely identifies previously unexplored structural characteristics within the risk perception network, including: ① Core drivers (most influential nodes); ② Bridge nodes (connecting distinct cognitive/behavioral domains such as scientific knowledge and protective actions); ③ Behavioral-inhibition modules (network clusters potentially hindering mitigation efforts).

**Figure 1:**
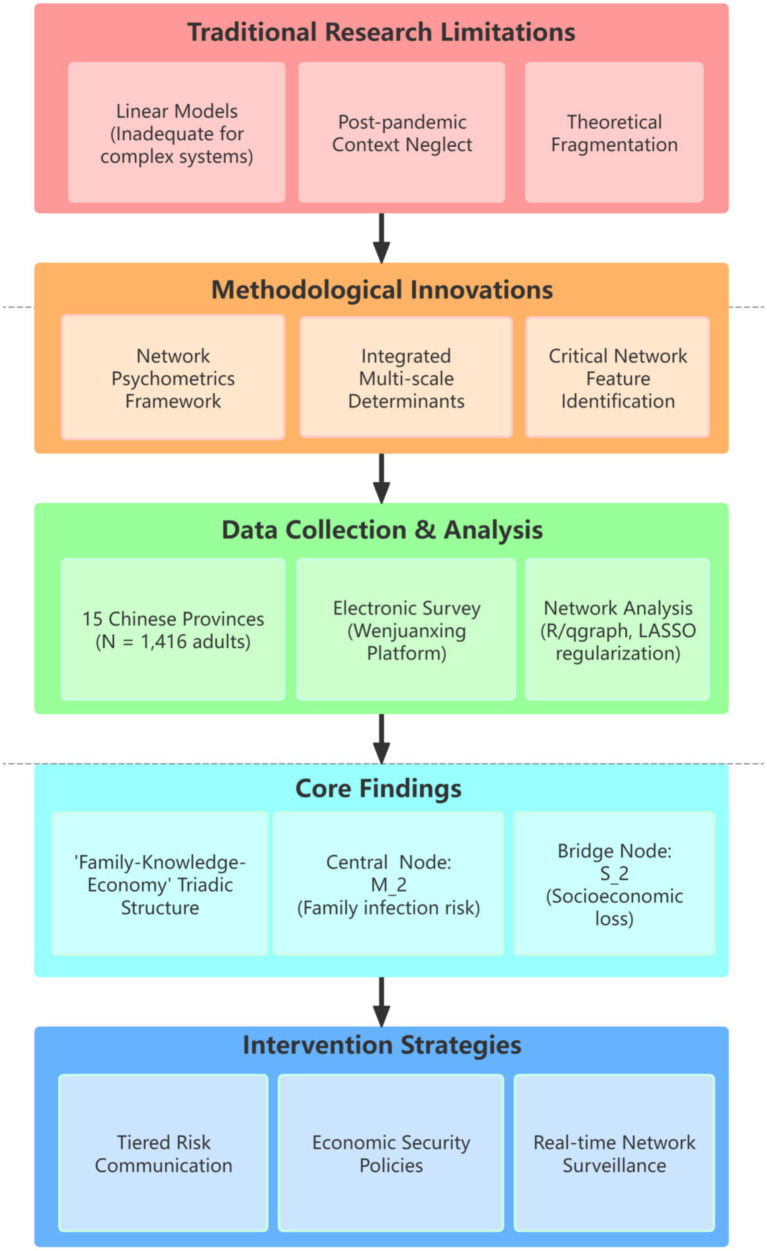
Research design.

These features provide novel targets for intervention. As illustrated in Figure 1, our research design specifically investigates: ① The core network structure of influenza risk perception among Chinese adults; ② Critical hubs (bridge nodes) linking scientific knowledge systems with behavioral domains (e.g., vaccination intent and hygiene practices); ③ Mechanisms of network reconfiguration driven by socioeconomic mediators (e.g., economic vulnerability) and environmental mediators (e.g., regional pollution levels).

Collectively, these innovations provide a causal framework for precision interventions. Our findings simultaneously account for individual cognition (25, 26), structural determinants (27, 28), and the pandemic-altered behavioral context, thereby advancing both theoretical foundations and practical implementation of effective influenza prevention strategies.

## Subjects and methods

### Study participants

This cross-sectional study employed convenience sampling was used to recruit from the general Chinese population (including public service workers, professionals, business personnel, agricultural workers, and students) between January 1, 2025 and March 1, 2025. Inclusion criteria were: ① Aged 18–70 years; ② Voluntary participation; ③ Ability to properly understand study materials and questionnaires; ④ Normal communication capacity. Exclusion criteria included: ① Unwillingness to cooperate; ② Diagnosis of severe mental illness or cognitive impairment. Elimination criteria comprised: ① Withdrawal during survey/experiment; ② Samples with ≥20% missing data; ③ A completion time <120 s or >30 min.

### Research instruments

The study utilized three assessment tools: a general information questionnaire, the Perceived Influenza Risk Scale (PIRS), and an influenza science knowledge awareness survey.

1. General Information Questionnaire
  Researcher-developed, collecting demographic data including age, gender, ethnicity, occupation, marital status, monthly household income per capita, and healthcare payment methods.
2. Perceived Influenza Risk Scale (PIRS)
  Developed by the research team based on previous experience (29), this 18-item scale assesses four dimensions: risk familiarity perception (6 items), risk controllability perception (4 items), risk severity perception (5 items), and risk susceptibility perception (3 items). Items employ a 5-point Likert scale (1 = “strongly disagree” to 5 = “strongly agree”), with total scores ranging up to 90 (higher scores indicate greater risk perception). Six senior experts (medicine, psychology, sociology) evaluated content validity, showing excellent results: item-level content validity index (I-CVI) ≥ 0.889 and scale-level content validity index (S-CVI) = 0.988. Internal consistency was strong (Cronbach’s *α* = 0.840 overall; subscales: risk familiarity *α* = 0.934, controllability *α* = 0.850, severity *α* = 0.903, susceptibility *α* = 0.978).
3. Influenza Science Knowledge Awareness Survey
  Two supplemental questions assessed participants’ and family members’ influenza knowledge: “How familiar are you with influenza-related scientific knowledge?” and “How familiar are your family members with influenza-related scientific knowledge?” Responses used a 5-point scale (1 = “not familiar at all” to 5 = “very familiar”).

### Survey methodology and quality control

#### Sample size calculation

This multicenter cross-sectional study complied with the STROBE (Strengthening the Reporting of Observational Studies in Epidemiology) guidelines for cross-sectional research (30). The sample size was determined using the standard formula for cross-sectional studies (31): n = [Z_(1-*α*/2) / *δ*]^2 × *p* × (1- *p*), where Z_(1-*α*/2) represents the standard normal distribution value corresponding to the confidence level. At *α* = 0.05, Z_(1-*α*/2) = 1.96; *p* denotes the estimated variation proportion, set to 0.5 to maximize sample size; and δ represents the acceptable margin of error, defined as 0.05 (5%). The theoretical sample size was calculated as 385 participants. To account for potential attrition during the study, an additional 10–20% sample size was added, resulting in a final sample size of ≥ 424 participants.

#### Pilot survey

Before the formal survey, this study determined the pre-survey sample size according to 10 to 30% of the total sample size (32). According to the calculation, the sample size of the formal survey was determined to be 462 people, so the pre-survey sample size should be 46 to 139 people. In this study, convenience sampling was used to select 50 members of the public in Sichuan Province for the pre-survey to test the clarity and appropriateness of the questionnaire. Based on the feedback from the pre-survey, the questionnaire content was modified and adjusted as necessary to ensure that the entries were accurate and easy to understand. The pretest results showed that the Cronbach’s *α* internal consistency coefficient of the PIRS scale was 0.874, and the *α* values of the subscales were risk familiarity (*α* = 0.936), risk controllability (*α* = 0.810), risk severity (*α* = 0.896), and risk susceptibility (*α* = 0.993), indicating that the scales had good internal consistency.

#### Formal survey

The formal survey was administered electronically via Wenjuanxing (Doi: www.wjx.cn), a professional online questionnaire platform widely utilized in China for its operational efficiency, security, and anonymity. QR codes generated through the platform were distributed across institutional networks (school classes, hospital departments, community health groups) to recruit mainland Chinese residents. Upon accessing the questionnaire, participants encountered an introductory page detailing research objectives, completion guidelines, and privacy protection measures. Electronic informed consent was mandatory prior to survey initiation, with platform settings restricting submissions to one response per IP address.

#### Quality control

Ethical compliance was ensured through fully anonymized data collection, exclusion of personal identifiers, and encrypted data storage with access controls. The study protocol received approval from the West China Hospital Biomedical Ethics Committee (Approval No. 787/2021). Electronic questionnaires incorporated compulsory informed consent pages with automated recording of response times and IP addresses to ensure procedural standardization and traceability. Data quality control involved dual independent data cleaning by trained researchers, applying three exclusion criteria: ① Completeness: Questionnaires with >20% missing values; ② Consistency: ≥3 logical contradictions; ③ Validity: >80% repetitive responses. Discrepancies were resolved by senior researchers. Comprehensive data audit trails and quality control logs were maintained throughout data collection and analysis, strictly adhering to STROBE guidelines.

#### Statistical analyses

In this study, statistical analyses were conducted utilising R software (version 4.4.3). The analysis was primarily facilitated by the qgraph and bootnet packages, which were employed for network analysis. Initially, descriptive statistics were generated for all variables. Continuous variables were expressed as mean ± standard deviation, and categorical variables as frequencies and percentages. In order to assess the degree of variability in the items of the PIRS scale, standard deviation was used as an indicator of informativeness. Redundancy between items was checked to ensure that there was no excessive overlap between scale entries.

#### Estimation and validation of PIRS network structure

Network analysis is a data-driven approach capable of revealing independent relationships between variables and exploring interactions among multiple variables in complex systems (33). In this study, network analysis was employed to investigate the association patterns among the 18 influenza risk perception items in the PIRS scale and how they collectively influence public risk perception of influenza. The network structure was constructed and visualized using the qgraph package in R software, where partial correlation matrices were transformed into network graphs displaying only significant edges (*p* < 0.05). The PIRS network was built using LASSO-regularized partial correlation analysis (34), which reduces noise through regularization to generate a sparser network that highlights key associations among risk perception items. Model selection was optimized using the Extended Bayesian Information Criterion (EBIC) to ensure both model fit and parsimony (35, 36). Network visualization implemented the Fruchterman-Reingold algorithm (“spring” layout) (27), which positions nodes with greater influence (risk perception items) centrally and clusters strongly associated items in close proximity, thereby providing an intuitive representation of the relationships among risk perception items.

In the constructed network: ① Nodes represent the 18 risk perception items from the PIRS scale (e.g., “F_Are you familiar with the sources of influenza transmission?”); ② Edges denote partial correlation coefficients between two items after controlling for all other items; ③ Edge thickness reflects association strength: thicker edges indicate stronger associations, while thinner edges indicate weaker associations; ④ Edge color: green edges represent positive correlations (e.g., when two risk perception items increase simultaneously), while red edges represent negative correlations (e.g., when one risk perception item increases while another decreases).

This visualization approach effectively demonstrates the complex structure of public influenza risk perception. The network characteristics are characterized by three metrics: Strength, Bridge Strength, and Predictability (27, 37, 38). ① Node Strength is defined as the sum of absolute weights of all edges connected to a given node, reflecting the overall influence of an influenza risk perception item within the network. For example, this metric can determine whether “Influenza may cause severe socioeconomic losses” serves as a central component in public risk perception; ② Bridge Strength represents the sum of edge weights connecting a node to items across different dimensions, measuring its cross-dimensional influence (27). A practical example would be examining how “My family members may contract influenza” influences “I may contract influenza”; ③ Predictability indicates the degree to which a risk perception item can be predicted by other items in the network. For instance, it measures how susceptible “My family members may contract influenza” is to being influenced by other risk perception items (e.g., “I may contract influenza”).

To ensure the reliability of the network analysis results, we employed edge accuracy evaluation and centrality stability analysis to verify the accuracy and stability of the network (34). Edge accuracy was assessed using 95% nonparametric confidence intervals (CIs) derived from 1,000 bootstrap samples. Narrower CIs indicate more precise estimation of edge weights (39). Centrality stability was examined using the correlation stability coefficient (CS coefficient) for node strength. A CS coefficient above 0.25 indicates acceptable results, while values exceeding 0.5 are considered ideal (36). For comparisons of edge weights and node strength, bootstrap CIs were utilized for difference testing. Statistical significance was determined when the CIs excluded zero.

#### Association between risk perception and influenza knowledge

To identify which PIRS items were most strongly associated with public influenza knowledge, we integrated an “Influenza Knowledge Awareness” node into the existing PIRS network structure. Regularized partial correlation analysis consistent with the LASSO methodology was applied to estimate association strengths between the knowledge node and each PIRS item, maintaining methodological consistency with the primary network estimation to ensure robust and parsimonious results (34). The resultant edge weights, visualized through network graphs, quantitatively represent the association strength between influenza knowledge and each risk perception item, thereby revealing the most knowledge-relevant components of public risk perception.

## Results

### Sample characteristics and demographic analysis

A total of 1,577 questionnaires were collected, yielding a 100% response rate. After rigorous quality control, 161 invalid questionnaires were excluded (55 from respondents aged <18 or >70 years, 20 with illogical responses, and 86 with completion times <120 min), resulting in 1,416 valid questionnaires (validity rate: 88.63%, 1,416/1,577). The sample demonstrated strong representativeness, covering 15 provinces across five regions of mainland China: Northern China (Beijing, Jilin, Liaoning), Southern China (Guangdong, Fujian, Yunnan), Eastern China (Jiangxi, Shandong, Zhejiang), Western China (Gansu, Sichuan, Yunnan) and Central China (Henan, Hubei, Hunan). The demographic characteristics revealed.

An analysis of demographic characteristics reveals that the gender ratio is moderate, with 46.8% of the population identifying as male and 53.2% as female. The majority of the population falls within the 18–30 age bracket (68.3%), with 12.8% falling within the 31–40 age bracket. The ethnic composition of the population is predominantly Han Chinese (89.6%), with other ethnic groups accounting for 10%. The data indicates that 4% of the population is employed in the public sector, with 37.7% of jobs in this sector. The second largest sector is education, with 25.1% of the population employed as students, while 13.4% are employed in commercial services. The third largest sector is the tertiary education sector, with 20.5% of the population employed in vocational/technical education, and 42.8% of the population employed in bachelor’s degree programmes. The majority of the population (60.8%) is married, and the vast majority (94.1%) of the public reported no religious affiliation. The geographical distribution of the population is as follows: 47.7% of the population resided in the countryside, while 52.3% resided in towns. The majority (27.6%) of households reported an income between $4,000 and $6,000, followed by those with an income below $4,000 (21.5%) and above $12,001 (19.6%). The majority (79.4%) of the population reported having medical insurance, and 73.2% of the population reported previous experience of severe influenza (Table 1).

**Table 1:** Characteristics of the public (*N* = 1,416).

Characteristics	Frequency (*n*)	Percentage (%)
Gender		
Male	662	46.8
Female	754	53.2
Age (years)		
18 ~ 30	967	68.3
31 ~ 40	308	21.8
41 ~ 50	81	5.7
51 ~ 70	60	4.2
Ethnicity		
Han	1,269	89.6
Others	147	10.4
Occupations		
Public service	534	37.7
Technical professionals	137	9.7
Commercial workers	190	13.4
Agricultural workers	126	8.9
Students	356	25.1
Others	73	5.2
Educational level		
Primary education or below	57	4
Secondary education	70	4.9
Vocational/Technical education	390	27.5
Bachelor’s degree	606	42.8
Master’s degree or above	293	20.7
Marital status		
Married	861	60.8
Single	555	39.2
Religious beliefs		
No	1,332	94.1
Yes	84	5.9
Household registration location		
Countryside	676	47.7
Town	740	52.3
Monthly income per capita		
Less than 2,000 RMB	304	21.5
2,000 RMB-4,000 RMB	391	27.6
4,001 RMB-6000 RMB	203	14.3
6,001 RMB-8000 RMB	128	9
8,001 RMB -10000 RMB	113	8
Above 10,001 RMB	277	19.6
Payment methods for medical activities		
Private paymentt	259	18.3
Medical insurance	1,124	79.4
Others	33	2.3
Have you ever had the flu		
Yes	1,036	73.2
No	380	26.8

Occupations were categorized into seven groups based on their primary work nature: public service (e.g., civil servants, healthcare workers, teachers), commercial workers (e.g., company employees, managers, self-employed individuals), other (retirement, etc). Single indicated separated, divorced, widowed, or never married, and married indicated married or partnered. “Yes” includes Buddhism, Christianity, Islam, Catholicism, Taoism.

### Measurement tools and core variable analysis

Table 2 presents the descriptive statistics (*Mean* ± *SD*) for the 18 items in the PIRS scale. Initial analysis confirmed data quality: ① All items demonstrated standard deviations within acceptable ranges; ② No low-information items were identified (i.e., *SD*s >2.5σ below the mean); ③ Inter-item correlations were <0.25, indicating no redundant items and confirming that each item independently measured distinct dimensions of public influenza risk perception. Additionally, the influenza-related knowledge assessment yielded the following scores: Public knowledge: 2.46 ± 1.10 and Family members’ knowledge: 1.95 ± 0.81.

**Table 2:** The Perceived Influenza Risk Scale (PIRS) and descriptive statistics for each risk perception question within the scale.

Items	Item description	Mean	SD
Risk familiarity			
F_1	(1) Are you familiar with the infectious agents of influenza?	1.79	0.77
F_2	(2) Are you familiar with how the flu is spread?	1.61	0.62
F_3	(3) Are you familiar with how contagious the flu is?	1.69	0.69
F_4	(4) Are you familiar with who is susceptible to the flu?	1.79	0.80
F_5	(5) Are you familiar with the dangers of the flu?	1.64	0.66
F_6	(6) Are you familiar with flu protection?	1.59	0.62
Risk controllability			
C_1	(1) Do you think the flu is controllable?	1.95	0.63
C_2	(2) Do you think the danger posed by the flu is manageable?	2.19	0.81
C_3	(3) Do you think the duration of the flu is manageable?	2.51	0.98
C_4	(4) Do you think the flu is affecting a manageable amount of people?	2.17	0.83
Risk seriousness			
S_1	(1) Influenza can have serious physical and psychological effects on individuals	1.95	0.80
S_2	(2) Influenza can cause serious socio-economic losses	1.74	0.68
S_3	(3) Influenza can take a heavy financial toll on families	1.89	0.74
S_4	(4) The flu can threaten my health and life and that of my family	1.91	0.79
S_5	(5) The flu can affect my daily life and that of my family	1.86	0.74
Risk munity			
M_1	(1) I might get the flu.	3.19	1.15
M_2	(2) My family could get the flu.	3.2	1.14
M_3	(3) My relatives and friends could get the flu.	3.12	1.12

F, risk familiarity; C, risk controllability; S, risk seriousness; M, risk munity.

### PIRS network characteristics analysis

#### Network structure features

**Figure 2:**
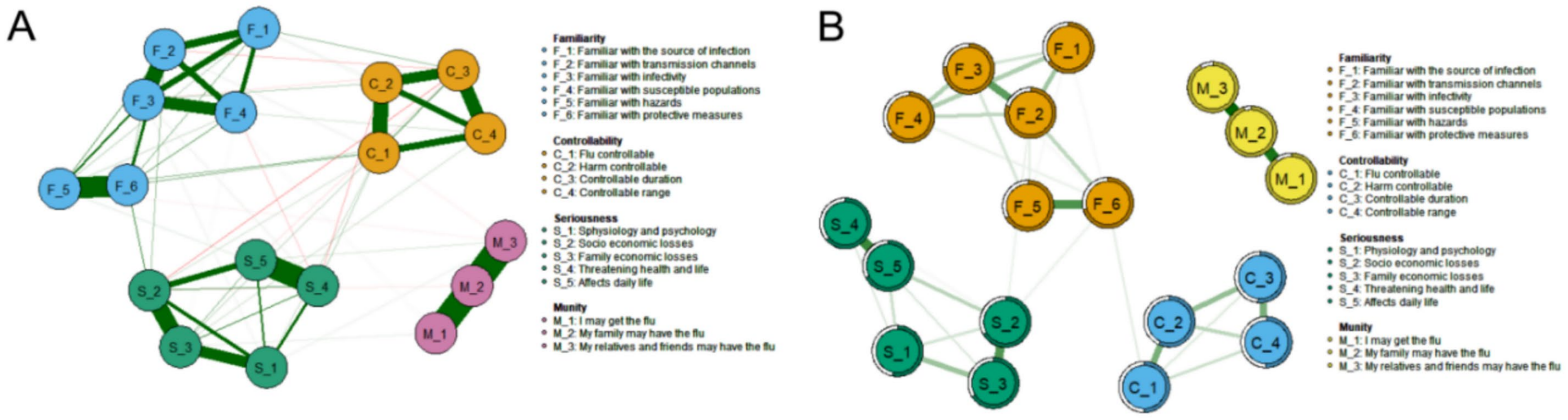
Influenza Risk Perception network diagram (*N* = 1,416). **(A)** Influenza risk perception network structure of 18 nodes, constructed using the graph package based on a partial correlation matrix. **(B)** Influenza risk perception network structure and predictability of the 18 nodes.

Figure 2A presents the network structure of the PIRS scale, comprising 18 nodes representing influenza risk perception items across four dimensions: Risk Familiarity (F_1-F_6), Risk Controllability (C_1-C_4), Risk Severity (S_1-S_5), and Risk Susceptibility (M_1-M_3). The network contained 73 non-zero edges (47.71% of possible edges, 73/153) with a mean edge weight of 0.054, indicating moderate-to-strong inter-item associations. Edges were derived from graphical partial correlation matrices, displaying only significant correlations. Green edges represent positive correlations, while red edges indicate negative correlations, with edge thickness and color intensity reflecting association strength.

#### Strength centrality

**Figure 3:**
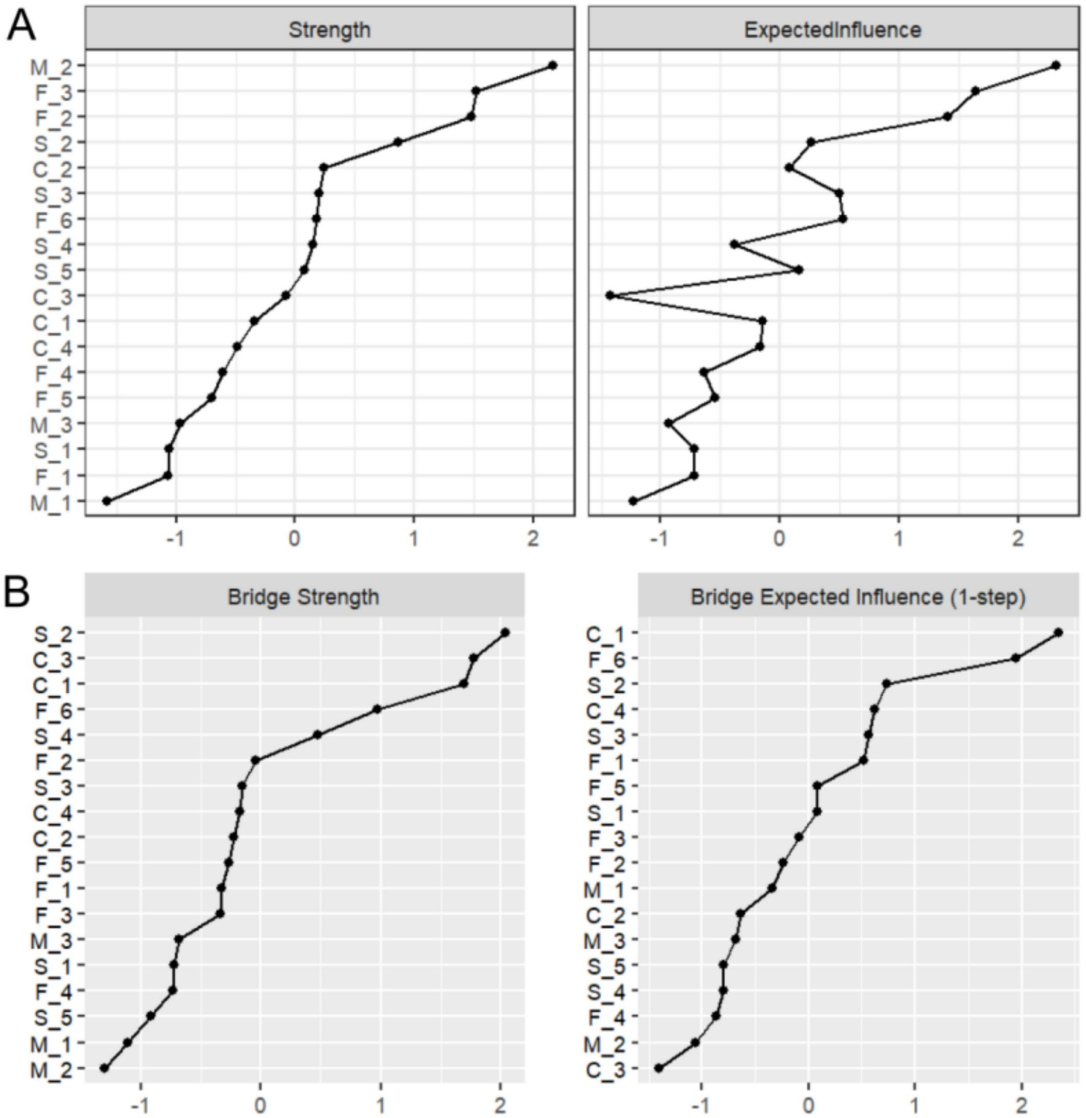
Results of strength and bridge strength analysis. **(A)** Strength centrality and expected influence for the 18 nodes in the network. **(B)** Bridge strength centrality and bridge expected influence for the 18 nodes in the network.

As shown in Figure 3A, strength centrality and expected influence were analyzed for all 18 nodes. The susceptibility item “M_2: My family members may contract influenza” demonstrated the highest strength centrality (Strength = 2.165), indicating its central role in the PIRS network and predominant influence on public risk perception. This was followed by familiarity items “F_3: Are you familiar with influenza infectivity?” (Strength = 1.520) and “F_2: Are you familiar with transmission routes?” (Strength = 1.478).

#### Bridge strength

Figure 3B displays bridge strength and bridge expected influence analysis results. The severity item “S_2: Influenza may cause severe socioeconomic losses” exhibited the highest bridge strength (Bridge Strength = 2.037), suggesting its crucial role in connecting different risk perception dimensions. Controllability items “C_3: Do you believe influenza duration is controllable?” (Bridge Strength = 1.774) and “C_1: Do you believe influenza is controllable?” (Bridge Strength = 1.691) ranked second and third, respectively.

#### Node predictability

Node predictability is visualized in Figure 2B through concentric circles, ranging from 50.5 to 95.2% (Table 3). The susceptibility item “M_2” showed the highest predictability (R^2^ = 0.952), indicating 95.2% of its variance could be explained by other network items. This was followed by “M_3: My relatives/friends may contract influenza” (R^2^ = 0.916) and “M_1: I may contract influenza” (R^2^ = 0.893) in the susceptibility dimension.

**Table 3:** List of nodes, their predictability, and their centality estimation.

Nodes	Variables	Predictability (R2)
F_1	Familiar with the source of infection	0.652
F_2	Familiar with transmission channels	0.798
F_3	Familiar with infectivity	0.765
F_4	Familiar with susceptible populations	0.635
F_5	Familiar with hazards	0.671
F_6	Familiar with protective measures	0.706
C_1	Flu controllable	0.502
C_2	Harm controllable	0.566
C_3	Controllable duration	0.516
C_4	Controllable range	0.505
S_1	Sphysiology and psychology	0.534
S_2	Socio economic losses	0.670
S_3	Family economic losses	0.657
S_4	Threatening health and life	0.637
S_5	Affects daily life	0.671
M_1	I may get the flu	0.893
M_2	My family may have the flu	0.952
M_3	My relatives and friends may have the flu	0.916
F_1	Familiar with the source of infection	0.652

F, risk familiarity; C, risk controllability; S, risk seriousness; M, risk munity.

### Accuracy and stability analysis of PIRS network

#### Edge weight accuracy

**Figure 4:**
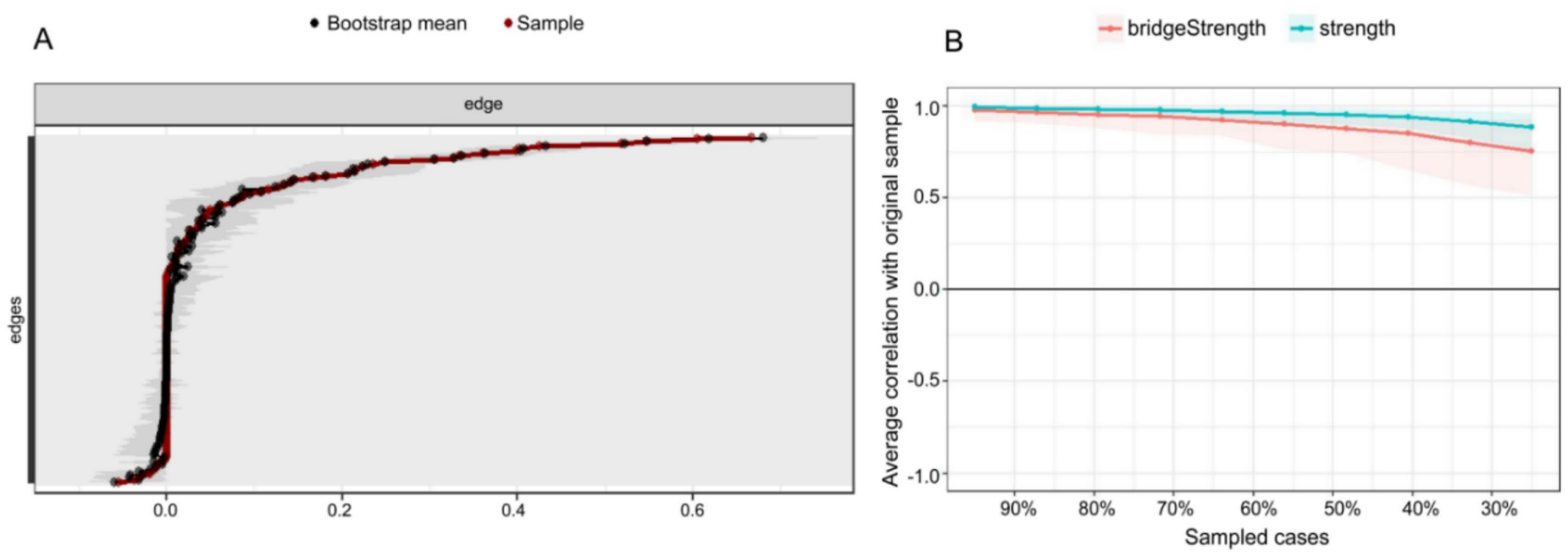
Accuracy and stability analysis of the influenza risk perception network. **(A)** Accuracy analysis of the edge weights. **(B)** Stability analysis of the centrality indices.

Figure 4A presents an accuracy analysis of edge weights in the PIRS network, where the 95% nonparametric confidence intervals (CIs) of the edge weights were calculated through 1,000 bootstrap samples. The results demonstrate that the average CI range for network edge weights was [0.0256, 0.0838], with a median connection strength of 0.0544, indicating moderate connectivity characteristics across the network. Specifically, most edge connections exhibited relatively narrow CIs (e.g., an average interval width of 0.0582), confirming high precision and stability in the estimation of core network parameters. However, it is noteworthy that some edges approaching zero showed wider CIs (interval width > 0.1), suggesting certain uncertainties in the estimation of these weaker connections. This finding not only validates the reliability of the PIRS network’s core structure but also provides important statistical reference for interpreting secondary associations within the network. We recommend further validation of these edge connections with larger samples in subsequent studies.

#### Centrality stability

As illustrated in Figure 4B, the stability analysis of strength centrality and bridge strength is evaluated using the correlation stability (CS) coefficient. The findings indicate that the CS coefficient for strength centrality attained 0.750, while bridge strength achieved 0.517, both surpassing the recommended threshold of 0.5 (36). This finding suggests that the centrality indices maintain high stability even when sample sizes are reduced, thereby indicating the robustness of the network analysis outcomes.

#### Within-network variance test

**Figure 5:**
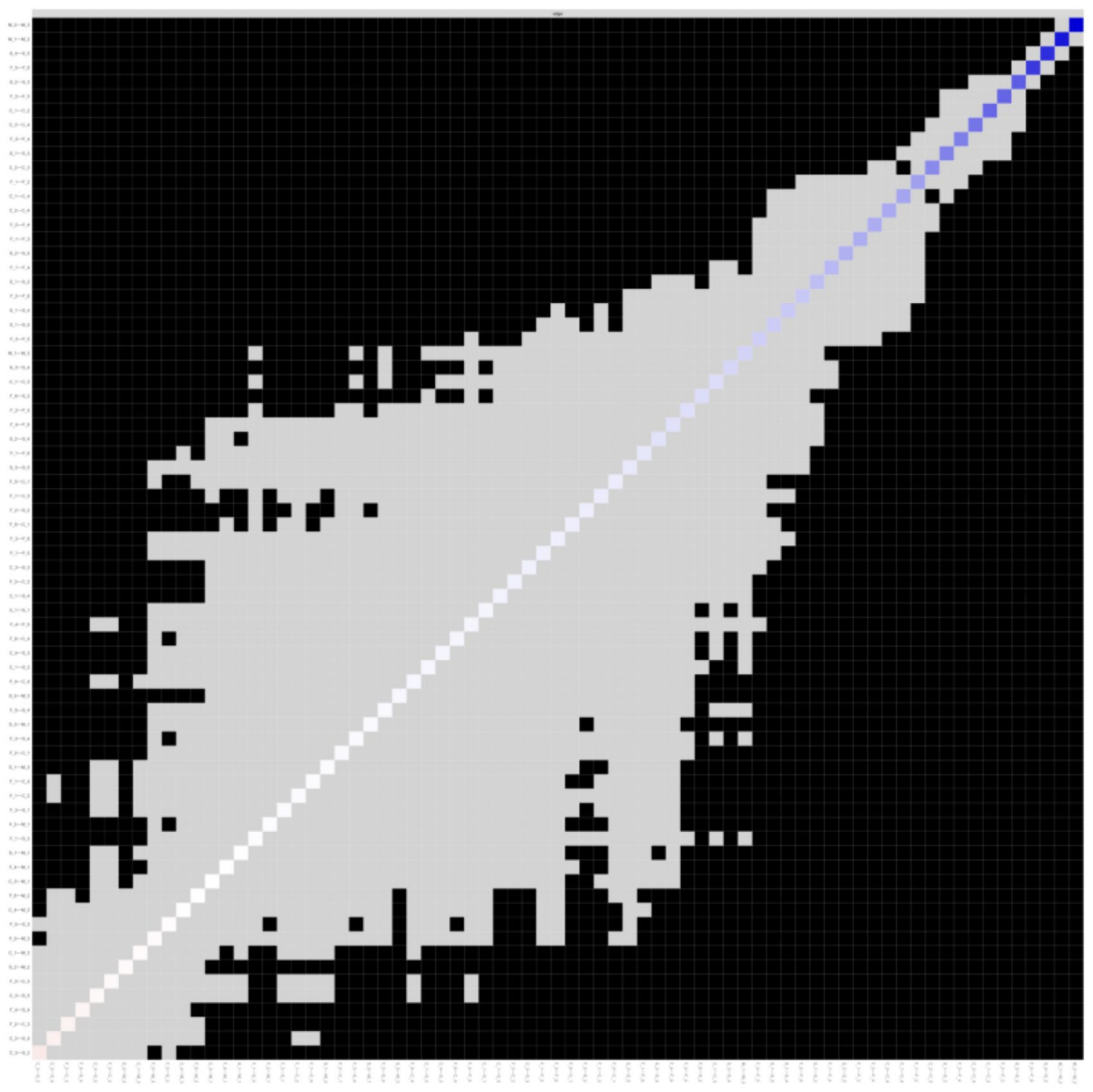
Bootstrapped difference test of edge weights. The x-axis and y-axis represent individual edge within the PIRS network. Gray boxes indicate non-significant differences, while black boxes indicate significant differences.

**Figure 6:**
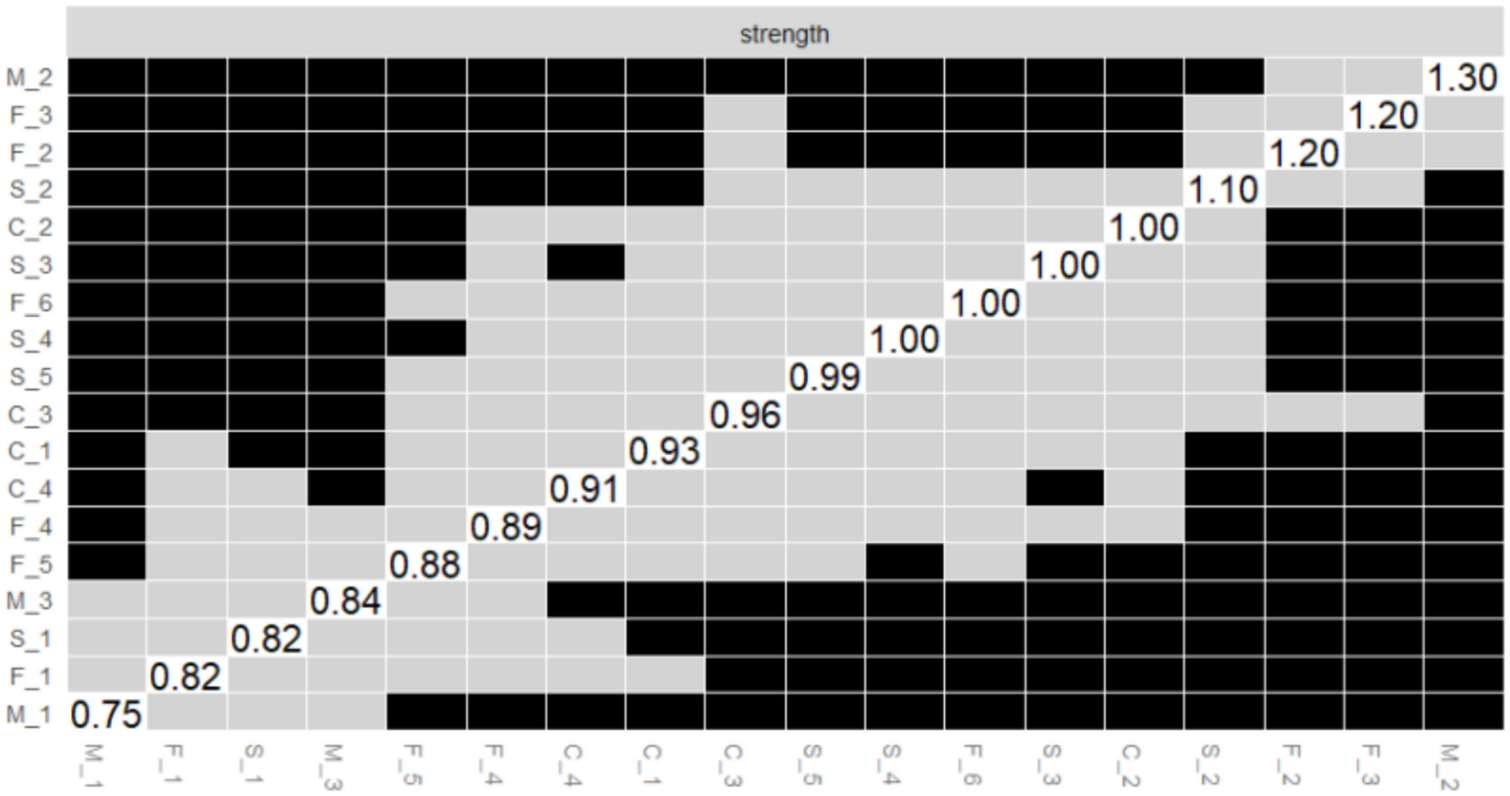
Bootstrapped difference test of the node strength centrality. The x-axis and y-axis represent individual nodes within the PIRS network. Strength centrality values are plotted on the diagonal. Gray boxes indicate non-significant differences, while black boxes indicate significant differences.

**Figure 7:**
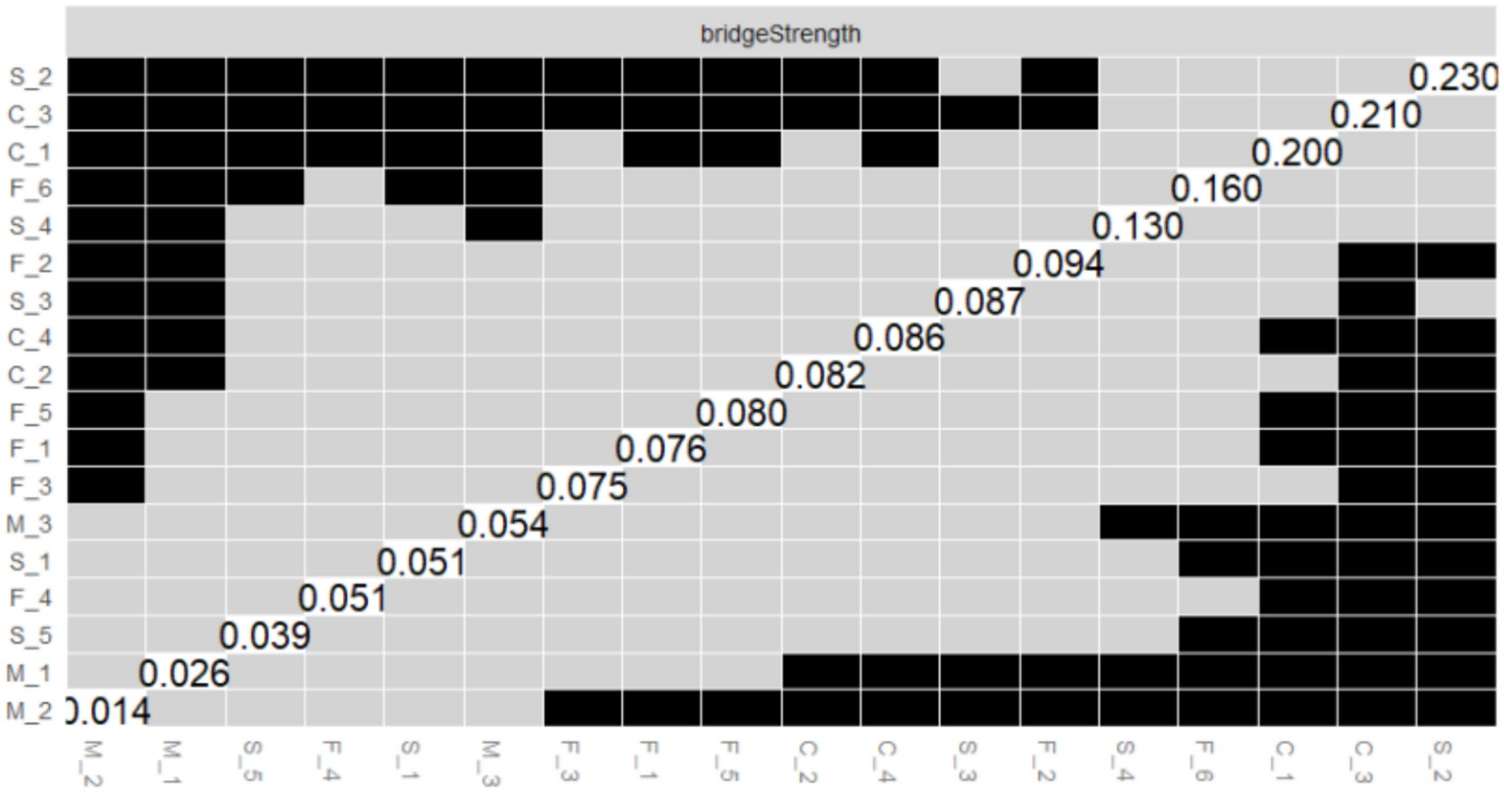
Bootstrapped difference test of the node bridge strength centrality. The x-axis and y-axis represent individual nodes within the PIRS network. Node bridge strength values are plotted on the diagonal. Gray boxes indicate non-significant differences, while black boxes indicate significant differences.

Figures 5–7 illustrate the results of the comparison of edge weights, node strength centrality and bridge strength centrality in the PIRS network using the bootstrap difference test.

#### Edge weight variance tests

As shown in Figure 5, this study systematically evaluated the edge weights of the PIRS network through bootstrap difference testing. Matrix analysis revealed that the majority of edge connections in the network exhibited significant differences (black squares > 65%), indicating high stability in network parameter estimation. Notably, the strongest connection strength was observed between M_2 (“My family members might contract the flu”) and M_3 (“My relatives or friends might contract the flu”) in the risk susceptibility dimension (edge weight = 0.667, 95% CI [0.609, 0.751]). This significant association (*p* < 0.001) reflects participants’ pervasive concern regarding influenza risk.

Additionally, stable moderate-strength connections were identified, such as M_1–M_2 (0.605, 95% CI [0.544, 0.693]) and S_4–S_5 (0.546, 95% CI [0.487, 0.610]). These findings collectively reveal core structural characteristics of influenza risk perception, where concerns about close interpersonal relationships constitute the most stable connections in the network. This provides critical evidence for developing targeted health education strategies for influenza prevention.

A small number of non-significant edges (gray squares) were observed, primarily distributed among secondary cross-dimensional connections, suggesting relatively weaker stability in these associations.

#### Node strength centrality difference test

As illustrated in Figure 6, the outcomes of the bootstrap difference test for node strength centrality (Strength) are presented. The matrix diagonal displays the strength centrality values for each node, with grey boxes denoting non-significant differences and black boxes indicating significant differences. The results demonstrate that M_2 (Strength = 2.165, see Figure 2A) in the risk susceptibility dimension and F_3 (Strength = 1.520, see Figure 2A) in the risk familiarity dimension exhibit significant differences from the majority of other nodes, with a greater number of black boxes in their corresponding rows. This finding serves to further validate the pivotal role of M_2 and F_3 in the PIRS network, thereby suggesting that the public’s perception of risk regarding the potential contraction of influenza by their family members, coupled with their familiarity with the contagious nature of the virus, exerts a substantial influence on the network dynamics.

As illustrated in Figure 7, the results of the bootstrap test of variance for node Bridge Strength are presented, and the matrix structure is analogous to that of Figure 6. The results demonstrate that S_2 (Bridge Strength = 2.037, see Figure 2B) in the risk severity dimension and C_3 (Bridge Strength = 1.774, see Figure 2B) in the risk controllability dimension exhibit significant differences from the majority of the other nodes, with the black boxes predominating in their respective rows. This finding indicates that S_2 and C_3 play a pivotal role in the network structure, facilitating connections between different dimensions of risk perception (e.g., risk severity vs. risk controllability). This observation underscores the significance of the public’s perceptions regarding the potential economic losses caused by influenza and the perceived manageability of the influenza duration within the network structure.

The results of the aforementioned difference-in-difference tests provide substantial support for the accuracy of the PIRS network estimates, indicating that the differences in the centrality and bridging roles of the core nodes (e.g., M_2, F_3, S_2, and C_3) in the network are statistically significant. These nodes represent the public’s risk perceptions that their family members may contract influenza, that they are familiar with the contagiousness of influenza, that influenza can cause severe socioeconomic losses, and that influenza lasts for a manageable period of time, respectively. This highlights the critical position of these risk perception items in the structure of the public’s perception of the risk of influenza, and provides important references for subsequent interventions (e.g., influenza science awareness design and application).

#### Associations between risk perception and knowledge of influenza science

**Figure 8:**
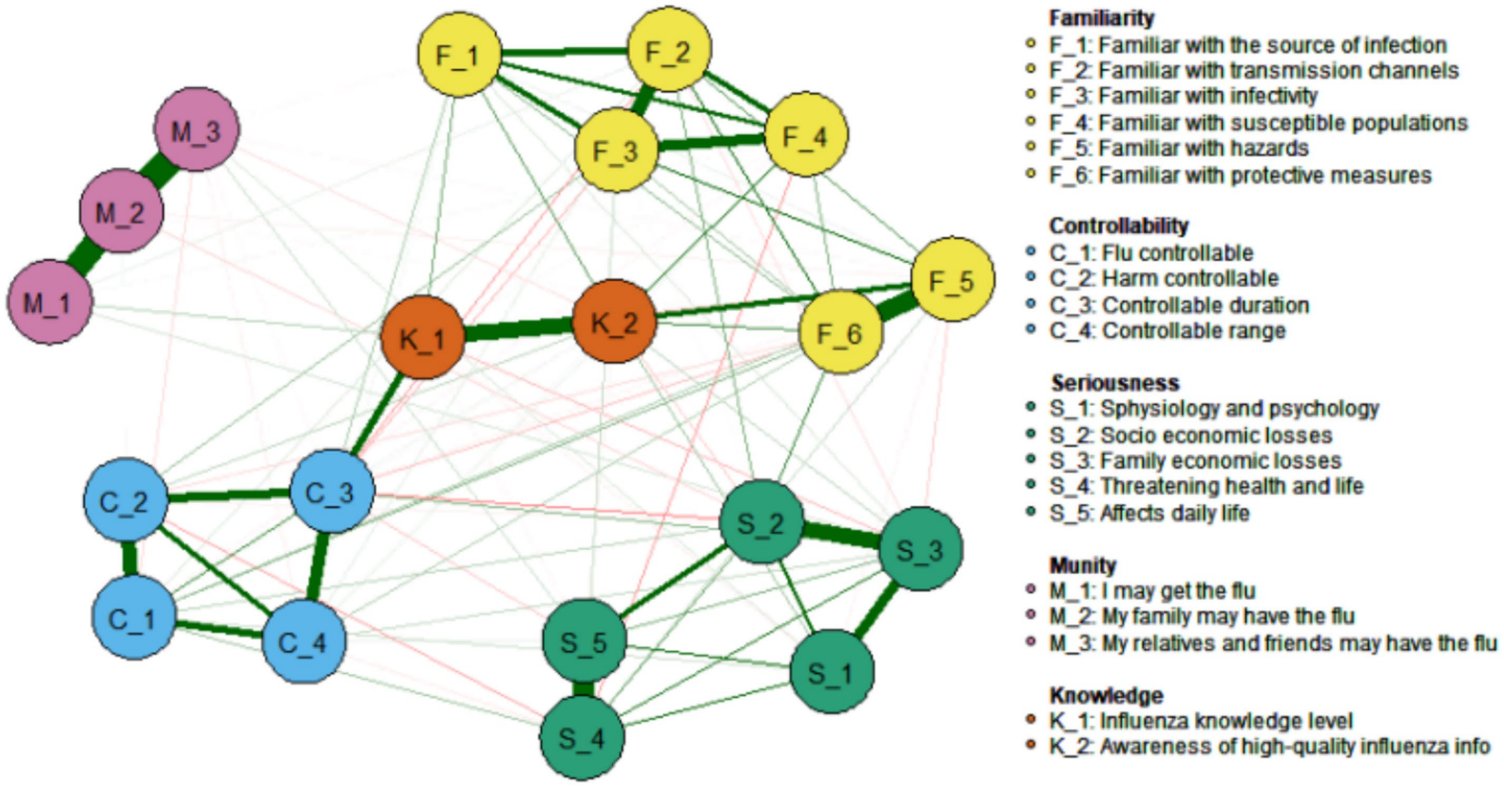
Associations between influenza risk perceived items and knowledge of science and technology.

As illustrated in Figure 8, the network correlation analysis reveals a relationship between the 18 risk perception items in the PIRS scale and two distinct dimensions of knowledge: public knowledge of influenza science (Knowledge, K_1) and family knowledge of influenza science (Knowledge, K_2). The results demonstrated that C_3 (the extent to which respondents deemed the duration of influenza to be manageable) and K_1 (public knowledge of influenza) of the risk controllability dimension exhibited the strongest association (Edge Weight = 0.25), followed by F_5 (respondents’ familiarity with the dangers of influenza) and K_2 (family knowledge of influenza) of the risk familiarity dimension (Edge Weight = 0.21). Meanwhile, K_1 (public knowledge of influenza) and K_2 (family knowledge of influenza) of the influenza knowledge dimension also demonstrated a strong association (Edge Weight = 0.42).

## Discussion

### Post-pandemic influenza risk perception: redefining vulnerability in the twindemic era

The COVID-19 pandemic has fundamentally altered influenza risk cognition patterns. Our network analysis reveals a collectivized family-knowledge-economy structure that differs markedly from pre-pandemic individual-centric models (40). Three paradigm shifts emerge.

#### Collectivization of fear

Household infection risk (M_2, Strength = 2.165) dominates the network topology, reflecting familial altruism rooted in sociocultural contexts. This aligns with global observations of immunity debt while contrasting with Western models emphasizing personal susceptibility (41, 42).

#### Economization of risk

Socioeconomic loss (S_2) emerged as the strongest bridge node (Bridge Strength = 2.037), demonstrating how pandemic-induced financial insecurity amplifies perceived influenza severity - a dimension overlooked in classical Health Belief Models (43). This finding corroborates studies linking economic vulnerability with vaccine hesitancy (44).

Knowledge-control paradox: While scientific literacy enhanced perceived controllability (C_3-K_1 edge = 0.25), it showed negligible impact on economic anxiety (S_2-K_1 edge = 0.08), explaining persistent vaccine hesitancy despite education campaigns. This aligns with research showing knowledge alone cannot overcome structural barriers to vaccination (44).

#### Theoretical implications

This triadic structure challenges linear knowledge-deficit models, positioning family protection motivation and structural vulnerability as co-determinants of risk perception - a critical advancement for collectivist societies (40).

### Network psychometrics: decoding complexity through systemic modeling

Our network analytic approach addresses three key limitations in risk perception research.

First, the LASSO-regularized partial correlation model (EBIC tuning = 0.5) uncovered non-linear pathways obscured by traditional methods (45). It revealed how transmissibility knowledge (F_3) amplifies family infection fear (M_2, edge weight = 0.38), which then exacerbates socioeconomic concerns (S_2, edge weight = 0.29) - a feedback loop explaining intervention failures. This systemic perspective aligns with findings that cognitive and affective risk dimensions interact dynamically (46).

Second, we identified three high-leverage node types with distinct intervention functions:

Core drivers (e.g., M_2: Family infection risk, strength = 2.165) serve as network anchors, consistent with studies showing kinship concerns dominate health decisions.

Knowledge amplifiers (e.g., F_3: Infectivity knowledge, strength = 1.520) function as information gateways, supporting targeted education strategies (47).

Structural bridges (e.g., S_2: Economic loss, bridge strength = 2.037) connect biological and material risks, necessitating integrated policies (44).

Third, the model quantified household-level diffusion dynamics, with the robust K_1-K_2 edge (weight = 0.42) confirming families as fundamental behavioral units. This finding extends neurocognitive evidence about kinship-based empathy while providing actionable precision for resource allocation (48).

Methodological validation: Our model showed superior predictive accuracy (centrality stability CS = 0.75) compared to linear approaches (mean R^2^ = 0.53), with bootstrap tests confirming node significance [ΔStrength (M_2 − F_3) = 0.645, *p* < 0.001]. Narrow confidence intervals for core edges [e.g., M_2-M_3: 95% CI (0.609, 0.751)] further validate its reliability (49). These psychometric properties align with recent advances in network medicine methodologies (50).

### Precision policy framework: from nodes to national strategy

Our findings necessitate a Precision Policy Framework that tailors interventions to specific network roles.

Firstly, tiered communication strategies must be implemented.

For critical bridge populations [e.g., low-income groups (monthly income <4,000 RMB; 21.5% of our sample)] and older adults [aged ≥60; high morbidity group underrepresented in our cohort (4.2%)], interventions should: ① Develop culturally sensitive narratives delivered via accessible channels (e.g., community theater, local radio, and social media platforms like Douyin/TikTok), focusing on economic risk mitigation (e.g., “Flu prevention protects your family’s income”) and vaccine cost subsidies (51); ② Distribute health kits containing rapid tests, masks, and pictorial educational materials in collaboration with neighborhood committees (52).

For core-driver groups [e.g., parents/caregivers (60.8% married in sample)], strategies include: ① School-based vaccination programs linked to incentives (e.g., childcare vouchers); ② Digital tools (WeChat mini-programs) providing family infection risk assessments and clinic locators.

Secondly, structural interventions must target key socioeconomic nodes, such as: ① Piloting flu-season income supplements for informal workers (e.g., 30% wage coverage during sick leave); ② Insurance premium reductions for vaccinated households (e.g., 5% discount on critical illness insurance) (53).

Finally, real-time surveillance of network dynamics through: ① Social media sentiment analysis (e.g., monitoring keywords: “flu costs,” “family infection fear”); ② Agent-based modeling simulating policy impacts on bridge nodes (e.g., economic loss perception) (54).

### The triadic model’s value for emerging “coinfection diseases”

The Triadic Model demonstrates considerable potential for addressing emerging challenges in coinfection diseases, particularly in the context of concurrent influenza and COVID-19 infections (often referred to as “twindemic” scenarios) (55). This framework offers distinct advantages over conventional linear models by effectively capturing the complex interplay of risk perception and behavioral responses inherent in coinfection situations (56).

The model’s strength lies in its ability to analyze how individuals simultaneously evaluate multiple pathogen threats, including assessments of susceptibility, severity, and controllability - often while navigating overlapping symptoms and conflicting public health information (57). Through its network structure, the model identifies critical drivers (e.g., persistent family infection concerns) and key bridging elements (e.g., heightened socioeconomic loss anxieties), revealing dynamic interactions between perceptions of different pathogens within the triadic structure (58).

Notably, the model highlights important behavioral insights, such as how scientific literacy may enhance perceived disease controllability without necessarily alleviating economic anxieties - a crucial consideration for intervention design (59). This finding underscores a critical gap in current approaches: effective coinfection management requires not only integrated disease knowledge but also targeted strategies to address compounded socioeconomic concerns that may impede adherence to complex mitigation protocols.

For enhanced predictive capability in coinfection research, future model iterations should incorporate several key expansions: ① Coinfection-specific risk perception metrics (assessing combined susceptibility/severity); ② Protective behavior nodes (e.g., dual vaccination uptake, combined protocol adherence); ③ Information complexity parameters (difficulty distinguishing diseases/accessing unified guidance); ④ Healthcare system strain indicators.

These enhancements would enable researchers to simulate how targeted interventions propagate through the network system, ultimately informing more effective public health strategies for concurrent epidemic scenarios (60). The model’s adaptability suggests particular promise for investigating behavioral dynamics surrounding emerging challenges like vaccination hesitancy and social distancing compliance in coinfection contexts (61).

### Global implications and future research

This framework holds significant global implications but requires contextual adaptation. Cross-cultural adjustments are essential; while our family-centric model proved effective, its application in individualistic societies may require prioritizing different network clusters (62). Integrating environmental risk factors is critical for regions facing compound health crises (63).

Future research should track long-term network changes to disentangle COVID-19 effects from structural shifts (64). Scalable intervention platforms represent a key pathway, including developing mobile apps with gamified education and establishing network response teams trained in targeted outreach.

## Limitations and future research directions

While this study provides valuable insights, several limitations warrant attention. First, the cross-sectional design precludes causal inference, and the network structure may vary dynamically with influenza epidemic cycles. Future research should adopt longitudinal designs to examine network evolution during pandemic fluctuations, incorporate neuroimaging techniques (e.g., fMRI) to investigate the neural mechanisms of risk assessment, and ultimately develop AI-powered dynamic risk early-warning systems. Despite these methodological constraints, our findings establish an important theoretical framework and intervention targets for post-pandemic influenza prevention, which should be empirically validated through randomized controlled trials.

Second, the sample exhibits significant demographic imbalances that may limit generalizability. Specifically: ① Age distribution was skewed toward younger adults (68.3% aged 18–30), with severe underrepresentation of older populations (only 4.2% aged 51–70). This may weaken the validity of findings for older adult subgroups who face higher influenza morbidity/mortality risks; ② Low-education groups were insufficiently captured (only 4% with primary education or below), potentially overlooking socioeconomic vulnerabilities linked to health literacy; ③ The convenience sampling approach and online questionnaire delivery may have excluded digitally disadvantaged populations (e.g., rural older adult, low-income households without internet access), introducing selection bias.

Consequently, policy implications—particularly those advocating family-centered interventions—should be interpreted with caution regarding generalizability to older adults and marginalized communities. Future studies must prioritize probability sampling (e.g., stratified random sampling) with quotas for underrepresented groups and employ mixed-mode surveys (online + community-based interviews) to enhance coverage.

Finally, the data collection approach may introduce measurement bias. The use of voluntary online self-report questionnaires risks social desirability bias and selection bias. Notably, the absence of standardized assessment tools for science knowledge measurement may affect the accuracy of network analysis results. Future research should combine subjective and objective measures (e.g., standardized tests, behavioral experiments) and adopt mixed-methods approaches (qualitative interviews + quantitative surveys) for triangulation validation. We also recommend developing cross-culturally adapted influenza knowledge assessment scales to enhance ecological validity.

## Conclusion

In this study, innovative network analysis methods were applied to reveal the triad driving mechanism of influenza risk perception among the Chinese public. The study found that family centrality and the fear of family members contracting influenza (M_2) were the core drivers of the risk perception network, highlighting the key role of the “family protection motive” in a collectivist culture. The findings of the study demonstrated a robust correlation between the awareness of infectiousness (F_3) and socioeconomic concerns (S_2), thereby underscoring the necessity for science education to encompass both biological perceptions and economic risk communication. Furthermore, the study identified a positive feedback loop between knowledge and sense of control, thus providing a precise target for “network interruption” interventions. In terms of practical implications, the following recommendations are made: the development of family-centred mitigation strategies, including the incorporation of vaccination reminders and financial compensation policies into family health kits; the design of tiered communication materials that emphasise data evidence for the highly educated and narrative case studies for the disadvantaged; and the establishment of a dynamic monitoring system to warn of sudden changes in risk perceptions using cyberindicators. This study innovatively applied network science to the study of influenza risk perception, providing new ideas for improving public health risk perception and optimising public health intervention strategies. It is imperative that future studies focus on the further optimisation of the network intervention model through the implementation of longitudinal designs and multi-country validation.

## Data Availability

The datasets presented in this study can be found in online repositories. The names of the repository/repositories and accession number(s) can be found in the article/supplementary material.
